# Antiangiogenic Therapy for Patients with Recurrent and Newly Diagnosed Malignant Gliomas

**DOI:** 10.1155/2012/193436

**Published:** 2011-07-14

**Authors:** Katsuyuki Shirai, Michael R. Siedow, Arnab Chakravarti

**Affiliations:** Department of Radiation Oncology, The Ohio State University Comprehensive Cancer Center, Columbus, OH 43210, USA

## Abstract

Malignant gliomas have a poor prognosis despite advances in diagnosis and therapy. Although postoperative temozolomide and radiotherapy improve overall survival in glioblastoma patients, most patients experience a recurrence. The prognosis of recurrent malignant gliomas is dismal, and more effective therapeutic strategies are clearly needed. Antiangiogenesis is currently considered an attractive targeting therapy for malignant gliomas due to its important role in tumor growth. Clinical trials using bevacizumab have been performed for recurrent glioblastoma, and these studies have shown promising response rates along with progression-free survival. Based on the encouraging results, bevacizumab was approved by the FDA for the treatment of recurrent glioblastoma. In addition, bevacizumab has shown to be effective for recurrent anaplastic gliomas. Large phase III studies are currently ongoing to demonstrate the efficacy and safety of the addition of bevacizumab to temozolomide and radiotherapy for newly diagnosed glioblastoma. In contrast, several other antiangiogenic drugs have also been used in clinical trials. However, previous studies have not shown whether antiangiogenesis improves the overall survival of malignant gliomas. Specific severe side effects, difficult assessment of response, and lack of rational predictive markers are challenging problems. Further studies are warranted to establish the optimized antiangiogenesis therapy for malignant gliomas.

## 1. Introduction

Malignant gliomas such as glioblastoma and anaplastic gliomas are the most common primary brain tumors in adults [1]. Temozolomide and radiotherapy have been demonstrated to improve overall survival in glioblastoma patients [2–4]. Despite advances in diagnosis and therapy, prognosis remains poor with a median overall survival of 12 to 15 months in glioblastoma due to the resistance to radiotherapy and chemotherapy. Although anaplastic gliomas tend to respond well to these treatments, the median survival time is 2 to 3 years [5, 6]. The prognosis of recurrent malignant gliomas is dismal with the median overall survival and progression-free survival (PFS) of 7.5 months and 2.5 months, respectively [7]. More effective therapeutic strategies are needed for these patients.

Malignant gliomas are characterized by vascular proliferation or angiogenesis [8, 9]. Vascular endothelial growth factor (VEGF) is highly expressed in glioblastoma and has been shown to regulate tumor angiogenesis [10]. Bevacizumab was developed as a humanized monoclonal antibody against VEGF. Clinical trials of recurrent glioblastoma showed benefits of bevacizumab in response rate and PFS [11–13]. Based on these favorable results, bevacizumab was approved by the US Food and Drug Administration (FDA) for recurrent glioblastoma. For newly diagnosed glioblastoma, phase II trials showed that the addition of bevacizumab to temozolomide and radiotherapy improves PFS [14, 15]. Other antiangiogenic drugs have also been investigated and used in several clinical studies [16]. In this paper, we focus on biological and clinical findings of antiangiogenesis therapy for malignant gliomas. 

## 2. Biological Aspects of Antiangiogenic Therapy for Glioblastoma

Advances in molecular biology have provided pathogenesis of malignant gliomas. Several clinical and preclinical studies proposed that tumor-related blood vessel, called “angiogenesis”, is required for solid tumor growth, including malignant gliomas [10, 16]. Endothelial proliferation is a marker of histological grading systems for malignant gliomas because of an association between a degree of microvascularity and biologic aggressiveness [17]. Glioblastoma is particularly characterized by vascular proliferation and the extent of necrosis. These findings indicate that tumor antiangiogenesis is a promising candidate to inhibit the growth of malignant gliomas. VEGF, a critical mediator of angiogenesis, has emerged as a novel target of antiangiogenic therapy. Glioblastoma cells have been shown to secrete VEGF, resulting in the endothelial proliferation and tumor survival *in vivo* [18]. VEGF is expressed in malignant gliomas and is associated with tumor grade and vascularity [19, 20]. Therefore, it is postulated that antiangiogenesis suppresses blood flow and inhibits the tumor growth. Monoclonal antibodies against VEGF were shown to inhibit the growth of glioma cells *in vivo* [21]. A VEGF inhibitor directly affects glioma stem cells that are more resistant to chemotherapy and radiotherapy [22]. Furthermore, antiangiogenesis can normalize tumor vasculature and decrease interstitial fluid pressure, providing an improved delivery of chemotherapeutics and oxygen. Consequently, antiangiogenesis is expected to work synergistically with radiotherapy and chemotherapy [23, 24]. Given these findings, VEGF inhibitors are expected to be a novel antiangiogenic therapy for malignant gliomas. 

## 3. The Efficacy and Safety of Bevacizumab for Recurrent Malignant Gliomas

### 3.1. Bevacizumab for Recurrent Glioblastoma

Bevacizumab was developed as a humanized monoclonal antibody to bind VEGF-A, preventing the interaction and activation of VEGF receptor tyrosine kinases [25, 26]. This drug is approved by the FDA and is in clinical use for the treatment of colorectal cancer, nonsmall cell lung cancer, breast cancer, renal cell carcinoma, and glioblastoma [27].  Table 1 shows several clinical studies of bevacizumab for recurrent malignant glioma patients.

The first phase II trial for 35 recurrent glioblastoma was performed to investigate the efficacy of intravenous administration of bevacizumab and irinotecan, a topoisomerase 1 inhibitor [11]. The 6-month PFS was 46%, and median overall survival was 10.5 months, respectively. At least a partial response was observed in 57% of patients. A larger, randomized, noncomparative phase II study, called the BRAIN study, was performed using bevacizumab with or without irinotecan for 167 recurrent glioblastoma. In this study, the response rates were 28.2% and 37.8%, and 6-month PFS were 42.6% and 50.3% in bevacizumab alone and bevacizumab plus irinotecan groups, respectively [12]. Another phase II study of bevacizumab alone for 48 recurrent glioblastomas showed that response rate was 35% and 6-month PFS was 29%, respectively [13]. These results were more favorable than a previous database of 8 negative trials having 6-month PFS of 15% for recurrent glioblastoma patients [7]. Furthermore, an additional advantage of bevacizumab is its ability to decrease tumor edema and to reduce steroid dose. Given the efficacy of bevacizumab for recurrent glioblastoma in the clinical setting, bevacizumab monotherapy has since been approved by the FDA. 

### 3.2. Toxicity Profile of Bevacizumab

Since VEGF plays an important role in vascular function and physiological angiogenesis, its inhibition via bevacizumab has been reported to cause serious adverse events [34]. The first phase II study of recurrent glioblastoma treated by bevacizumab and irinotecan reported that five patients (14%) discontinued treatment due to central nervous system (CNS) hemorrhage, deep venous thrombosis, and pulmonary emboli [11]. No fatal adverse events were reported in this study. BRAIN study reported the detailed information on adverse events of bevacizumab [12]. Grade ≥3 adverse events were observed in 65.8% and 46.4% of bevacizumab plus irinotecan and bevacizumab alone groups, respectively. The most common causes of grade ≥3 adverse events were convulsion, hypertension, neutropenia, and fatigue. CNS hemorrhages of any grades were observed in five patients (3.0%). There were one fatal adverse event (1.3%) in bevacizumab plus irinotecan group and two (2.3%) in bevacizumab alone group, respectively. Selecting appropriate patients, early assessment of toxicity, and adequate management should be required to reduce the serious adverse events of bevacizumab.

 Arterial and venous thromboses are generally reported in treatment of antiangiogenesis therapy, although the inherent risk of these thromboses is higher among malignant glioma patients. A retrospective study of 9849 patients with malignant gliomas showed that 2-year cumulative incidence of symptomatic venous thromboembolism was 7.5% [35]. Further studies are necessary to evaluate the additional risk of thrombosis in malignant glioma patients treated by bevacizumab.

Recently, a meta-analysis of randomized control trials in several tumor types showed that bevacizumab in combination with chemotherapy increases fatal adverse events when compared with chemotherapy alone [36]. The overall incidence of fatal adverse events was 2.5% in bevacizumab therapy with the common causes being hemorrhage, neutropenia, and gastrointestinal tract perforation. Interestingly, the type of chemotherapeutic agents was significantly associated with relative risk of fatal adverse events. The addition of bevacizumab was associated with increased fatal adverse events in patients receiving taxanes or platinum agents (3.3% versus 1.0%) but not in those receiving other agents (0.8% versus 0.9%). In clinical trials of malignant gliomas, the addition of irinotecan or temozolomide to bevacizumab has often been performed and may be associated with lower fatal adverse events. However, this meta-analysis did not include the trials of brain tumors, and further investigations are required to evaluate the fatal adverse events of bevacizumab and chemotherapy in malignant gliomas. 

### 3.3. Bevacizumab for Recurrent Anaplastic Gliomas

Anaplastic gliomas have a slightly better prognosis than glioblastoma [37], and the median survival time is 2 to 3 years. However, there are no standard treatments for progression or recurrence of anaplastic gliomas, and a novel treatment strategy is needed. Recent studies have shed light on the antiangiogenic therapy in recurrent anaplastic gliomas. A phase II study of bevacizumab and irinotecan was performed in 23 glioblastoma and 9 anaplastic gliomas [28]. The response rates were 61% and 67%, and 6-month PFS were 30% and 56% in glioblastoma and anaplastic gliomas, respectively. Another phase II trial was conducted for 33 anaplastic gliomas treated by bevacizumab and irinotecan [29]. This study included 25 anaplastic astrocytomas and 8 anaplastic oligodendrogliomas. The 6-month PFS and overall survivals were 55% and 79%, respectively. At least a partial response was observed in 61% of patients, and dose of dexamethasone was decreased in 67%. These findings indicate that bevacizumab and irinotecan can be an active regimen for recurrent anaplastic gliomas. 

### 3.4. Additional Treatment to Bevacizumab for Recurrent Malignant Gliomas

The addition of targeting therapy or radiotherapy to bevacizumab has been performed for recurrent malignant gliomas. Gutin et al. retrospectively analyzed bevacizumab and stereotactic radiotherapy (30 Gy in 5 fractions) for 25 recurrent malignant gliomas [30]. There were 20 glioblastomas and 5 anaplastic gliomas in this study with all patients receiving prior radiotherapy. Response rate was 50%, and 6-month PFS was 65% in glioblastoma patients. Three patients (12%) discontinued treatment due to tumor hemorrhage, wound dehiscence, and bowel perforation, although no radiation necrosis was detected. The authors concluded that treatment was well tolerated and beneficial for recurrent malignant gliomas. Cuneo et al. retrospectively evaluated the efficacy and safety of stereotactic radiosurgery and adjuvant bevacizumab for recurrent malignant gliomas [31]. Median PFS was 5.2 months, and 1-year overall survival was 50% in glioblastoma patients treated by radiosurgery and adjuvant bevacizumab. These results were significantly better than radiosurgery and other drugs. The authors concluded that salvage radiosurgery and bevacizumab improve outcomes in recurrent malignant gliomas.

A phase II study of bevacizumab plus erlotinib, an epidermal growth factor receptor (EGFR) tyrosine kinase inhibitor, was performed for patients with recurrent malignant gliomas [32]. This study included 25 glioblastomas and 32 anaplastic gliomas. The response rate and 6-month PFS were 48% and 28% for glioblastoma and 31% and 44% for anaplastic gliomas, respectively. Grade 1 or 2 rash, mucositis, diarrhea, and fatigue were the most common adverse events. The authors concluded that treatment was tolerated, but the additional benefits of erlotinib were unclear when compared with historical bevacizumab-containing regimens. These studies indicate that additional therapy to bevacizumab can be promising strategy, although it is still unclear which agent has the efficacy in combination with bevacizumab for malignant gliomas. Further studies are required to establish the additional agents to bevacizumab. 

## 4. Resistance to Bevacizumab

In the maintenance of bevacizumab, patients with malignant gliomas inevitably experience tumor recurrence. Furthermore, recurrent tumors after bevacizumab failure are reported to be more aggressive with rebound edema [38]. Although additional agents to bevacizumab have been attempted for patients after bevacizumab failure, disease prognosis was extremely poor with median PFS of 37.5 days and 6-month PFS of 2%, respectively [39]. The authors concluded that alternative strategies should be considered for these patients. De Groot et al. showed that bevacizumab induced a particularly invasive tumor phenotype expressing insulin-like growth factor binding protein-2 and matrix metalloprotease-2 in glioblastoma [40].

 Preclinical studies indicated that alternative pro-angiogenic signaling pathways are upregulated in resistance to antiangiogenic therapies [41]. These other angiogenic factors such as fibroblast growth factors and platelet-derived growth factors (PDGF) can compensate for the loss of VEGF activity under bevacizumab treatment [42]. Additional agents inhibiting other antiangiogenic pathways may suppress these resistances, and further clinical and animal studies are clearly required to overcome the resistance of bevacizumab. 

## 5. Imaging of Response to Antiangiogenic Therapy

Most studies defined partial and complete responses as radiological objective response according to McDonald criteria that are based on contrast-enhanced CT or MRI [43]. However, an accurate assessment of tumor response by conventional modality is limited, since bevacizumab directly alters tumor blood vessels [44]. As a result of this, response rate and 6-month PFS are debatable as a measure of antitumor activity [44]. Norden et al. reported that bevacizumab suppressed enhancing tumor recurrence, but not nonenhancing and infiltrative tumor growth, indicating that bevacizumab may change the recurrence patterns of malignant gliomas [45]. Iwamoto et al. reported that contrast-enhanced MRI did not adequately evaluate disease status, whereas nonenhancing tumor recurrence was significantly associated with overall survival in recurrent glioblastoma treated by bevacizumab [46]. Given these findings, The Response Assessment in Neuro-Oncology Working Group was established to develop the new response criteria for clinical trials of brain tumors [47]. They proposed to incorporate T2 and fluid-attenuated inversion recovery (FLAIR) changes on MRI to assess the infiltrative pattern progression of malignant gliomas.

Other studies have looked into establishing a reliable radiological modality in antiangiogenesis therapy. Positron emission tomography (PET) using [^18^F] fluorothymidine (FLT) offers noninvasive assessment of cell proliferation [48]. The response measured by FLT-PET significantly predicted the overall survival in recurrent glioblastoma treated by bevacizumab (*P* = 0.061) [49]. Recently, Ellingson et al. reported that relative nonenhancing tumor ratio, the ratio of FLAIR to contrast-enhancing volume, was predictive for overall survival and PFS in the treatment of bevacizumab for recurrent glioblastoma [50]. Further studies are warranted to establish the imaging modality to evaluate the response to antiangiogenic therapy and to predict the prognosis. 

## 6. Biological Markers Predicting Response

A variety of biomarkers predicting the efficacy of bevacizumab have been reported in several tumor types including malignant gliomas [51]. These predictive biomarkers are expected to lead to a personalized therapy that selects patients who can benefit from bevacizumab. Sathornsumetee et al. examined several biological markers in recurrent malignant gliomas treated by bevacizumab and irinotecan [52]. High VEGF expression was significantly associated with higher radiographic response (*P* = 0.024), and high carbonic anhydrase 9 expression predicted poor overall survival (*P* = 0.016). Higher hypoxia-inducible factor-2 alpha and VEGF receptor-2 expressions were also reported to be associated with poor survival in recurrent malignant gliomas treated by bevacizumab and erlotinib [32].

Recently, circulating VEGF concentrations are reported to predict the prognosis in solid tumors treated by bevacizumab [51, 53]. The measurement of circulating proteins is an attractive strategy since blood is easily accessible and the assay is inexpensive. Circulating VEGF concentrations are expected to reflect VEGF-dependent angiogenesis and to predict the benefit from bevacizumab [51]. Gururangan et al. examined the VEGFR-2 phosphorylation in peripheral blood mononuclear cells in recurrent malignant gliomas and diffuse brainstem glioma treated by bevacizumab [54]. They showed that circulating VEGFR-2 was inhibited by bevacizumab, but they did not show information on whether it is a prognostic biomarker. These clinical trials have provided some potential predictive markers (e.g. tumor VEGF expression or circulating markers), which require a phase III study for proper evaluation [55]. 

## 7. Addition of Bevacizumab to Temozolomide and Radiotherapy for Newly Diagnosed Glioblastoma

Several clinical studies have been performed to evaluate the safety and efficacy of the addition of bevacizumab for newly diagnosed glioblastoma (Table 1). Lai et al. reported a phase II study of the addition of bevacizumab to the standard treatment of temozolomide and radiotherapy for 70 newly diagnosed glioblastomas [14]. Bevacizumab was intravenously administered every 2 weeks from the first day of treatment. The median overall survival and PFS were 19.6 and 13.6 months, respectively. The authors concluded that the addition of bevacizumab improved PFS but not overall survival when compared with a control group treated with first-line temozolomide and radiotherapy who had mostly received bevacizumab at recurrence. Another phase II study also reported preliminary results on the addition of bevacizumab to the standard temozolomide and radiotherapy regimen in 125 newly diagnosed glioblastomas [15]. In this study, toxicity was minimal, and most patients (90%) continued treatment, with median PFS of 13.8 months. Recently, Desjardins et al. reported a phase II study of bevacizumab in combination with temozolomide plus radiotherapy followed by bevacizumab, temozolomide, and irinotecan for 125 newly diagnosed glioblastomas at the Society for Neuro-Oncology (SNO) annual meeting in 2010 [33]. This study had median overall survival of 21.3 months and PFS of 13.8 months, respectively. These studies showed encouraging results; however, it is still unclear whether the addition of bevacizumab to standard temozolomide and radiotherapy can improve the overall survival.

Currently, two randomized phase III trials, ROTG 0825 and AVAGLIO, are ongoing for newly diagnosed glioblastoma treated by temozolomide and radiotherapy with or without bevacizumab [56, 57]. These studies will show the role of bevacizumab in frontline treatment in glioblastoma patients. 

## 8. The Effect of Bevacizumab on Radiation Adverse Events

Bevacizumab has been reported to affect the specific adverse events of radiotherapy [58, 59]. Sherman et al. reported that six glioblastoma patients developed severe radiation optic neuropathy following bevacizumab [58]. All of them received 60 Gy in 30 fractions in the initial treatment. Patients received a median of 7.5 doses of bevacizumab followed by onset of visual symptoms. Although the detailed mechanism remains unclear, the authors indicated that bevacizumab decreases optic nerve tolerance to radiation. Another case series study reported that bevacizumab induced optic neuropathy and Brown-Sequard syndrome after irradiation [59]. The authors hypothesized that bevacizumab following radiotherapy inhibits VEGF-dependent repair of normal neural tissue.

In contrast, bevacizumab has been reported to be effective for the management of radiation necrosis and retinopathy [60, 61]. Radiation necrosis is a serious complication of radiotherapy and includes extended edema. Pathological findings show that endothelial cell dysfunction causes tissue hypoxia and necrosis with the local cytokine release, including VEGF [62, 63]. Corticosteroids, surgery, anticoagulation, and hyperbaric oxygen have been performed, although there is no evidence to support routine use in clinical practice [64]. Retrospective studies have shown that bevacizumab decreased the edema and improved the clinical outcome in patients with radiation necrosis [60, 65–67]. Interestingly, a small randomized trial was recently performed to demonstrate this effect [68]. Patients having radiation necrosis with progressive neurologic symptoms were assigned to bevacizumab (*n* = 14) and placebo groups (*n* = 7). Bevacizumab was intravenously administered every 3 weeks for 12 weeks. Radiological response and improvement of neurological symptoms were observed in the bevacizumab treated group but not in placebo group. The authors concluded that the class I evidence of bevacizumab efficacy for radiation necrosis was shown in this study.

Radiation retinopathy is a chronic and progressive condition that results from radiation exposure. Retinal vascular endothelial cell damage causes microaneurysms, telangiectasias, neovascularization, vitreous hemorrhage, macular edema, and tractional retinal detachment. Radiation retinopathy has been treated by laser photocoagulation, corticosteroids, and anticoagulation, although the management is still challenging [61]. Bevacizumab has been expected to be a therapeutic modality for radiation retinopathy. Finger reported that intravitreal injection of bevacizumab was effective for retinal hemorrhage, exudation, and edema, which improved visual acuity of patients [69]. There were no ocular and systemic side effects by bevacizumab. Furthermore, the authors recently showed that intravitreal bevacizumab was effective for radiation optic neuropathy [70].

Although these results indicate that some radiation vasculopathies are potentially treatable by bevacizumab, exacerbation of radiation necrosis by this drug was also reported [71]. It is still unclear how bevacizumab affects the radiation adverse events. Meticulous followup is required when bevacizumab is administered after radiotherapy. Animal models of radiation necrosis are needed to investigate the mechanism of bevacizumab. 

## 9. Other Antiangiogenic Drugs for Malignant Gliomas

VEGF has been shown to be the main player in tumor angiogenesis, and its inhibitor, bevacizumab, has been thoroughly investigated in clinical and animal studies. Other drugs such as pan VEGF receptor tyrosine kinase inhibitors have also been reported to inhibit VEGF pathways. Several biological pathways including integrin, fibroblast growth factor, and PDGF also are associated with the angiogenesis. Currently, several types of antiangiogenic drugs have been investigated and used in clinical trials for recurrent as well as newly diagnosed glioblastoma [72]. In this section, we review these drugs and the results of clinical trials (Table 2).

### 9.1. Cilengitide

Cilengitide competitively binds *αvβ*3 and *αvβ*5 integrin receptors that are expressed on tumor cells and activated endothelial cells during angiogenesis. Cilengitide can directly inhibit the growth of integrin-expressing tumor cells and indirectly act as an antiangiogenesis agent [87, 88]. Glioblastoma cells express integrin receptors, and cilengitide has shown an antitumor effect in glioblastoma xenografts *in vivo* [89, 90]. A randomized phase II study of 81 recurrent glioblastoma was performed to determine the efficacy and safety of cilengitide [73]. The patients were randomly assigned to receive either 500 or 2000 mg of cilengitide twice weekly. Patients treated with 2000 mg showed a trend toward better results with 6-month PFS of 15%. The treatment was well tolerated, and significant hematologic toxicity was uncommon. A phase I/IIa study of cilengitide combined with temozolomide and radiotherapy for 52 newly diagnosed glioblastoma patients was conducted [74]. This combination therapy was well tolerated without additional toxicity, and median overall survival was 16.1 months. The authors concluded that this regimen showed promising activity against newly diagnosed glioblastoma when compared with historical controls. Based on these results, two randomized trials, CENTRIC and CORE, are currently ongoing to determine the efficacy of cilengitide for newly diagnosed glioblastoma with or without a methylated O^6^-methylguanine-DNA methyltransferase (MGMT) promoter [91, 92]. 

### 9.2. Thalidomide and Lenalidomide

Thalidomide was developed as a sedative drug in 1950s and was withdrawn due to teratogenic effects. However, thalidomide was recently reported to have an antiangiogenic activity by inhibiting basic fibroblast growth factor (bFGF) [93], which can be exploited as an antitumor drug. Several clinical trials have been performed to assess the efficacy and safety of thalidomide for vascular tumors including malignant gliomas. This drug has since been approved by the FDA for the treatment of malignant myeloma [94]. Fine et al. showed a phase II study of thalidomide alone for 39 patients with recurrent malignant gliomas [75]. Thalidomide was well tolerated with modest sedation and constipation, although median PFS and overall survival were 2.5 months and 7.0 months, respectively. Another phase II study of thalidomide combined with carmustine was performed for 40 recurrent malignant gliomas [76]. Although the addition of carmustine seemed to improve the prognosis, the response rate and median PFS of combination group were 24% and 3.3 months, respectively. Puduvalli et al. reported a phase II trial of thalidomide and irinotecan for 32 recurrent glioblastomas [77]. The combination therapy was well tolerated with mild myelosuppression and sedation. At least a partial response was detected in two patients (6%), and 6-month PFS was 25%, respectively. These results indicate that thalidomide plus cytotoxic agents seem to have a mild antitumor activity for recurrent malignant gliomas patients when compared with thalidomide alone.

Lenalidomide, a potent structural and functional thalidomide analog, has antiangiogenic, anti-inflammatory, and immunomodulatory activities in preclinical studies [95, 96]. This drug is approved by the FDA for myelodysplastic syndrome with chromosome 5q deletion and multiple myeloma. Recently, lenalidomide has been performed for recurrent brain tumors in clinical trials [78, 97]. Fine et al. reported that lenalidomide was well tolerated; however, no objective responses were seen in a phase I study [78]. Median 6-month PFS was 12.5% in recurrent glioblastoma patients. Warren et al. conducted a phase I study of lenalidomide for pediatric patients with recurrent or progressive brain tumors [97]. This treatment was well tolerated with the primary toxicity being myelosuppression. Partial responses were seen in two patients (4%) with low-grade gliomas. Because these studies were phase I trials, further investigations are required to evaluate the antitumor activity for malignant gliomas. 

### 9.3. VEGF Receptor Tyrosine Kinase Inhibitors (Cediranib, Sorafenib, Sunitinib, Vatalanib, and Pazopanib)

Currently, VEGF receptor tyrosine kinase inhibitors are viewed as promising antiangiogenic agents in the setting of malignant gliomas. Cediranib was developed as an oral pan-VEGF receptor tyrosine kinase inhibitor. Preclinical studies showed that cediranib normalized tumor vasculature and decreased the edema in glioblastoma, improving the prognosis without inhibition of tumor growth [98, 99]. Batchelor et al. conducted a phase II study of cediranib for 31 recurrent glioblastoma patients [79]. Patients were administered a 45 mg/day dose of cediranib. Partial response according to the MacDonald criteria was observed in 26.6% of patients, and 6-month PFS was 25.8%. Corticosteroids were reduced or discontinued in 27% of patients. Toxicities were manageable, and common Grade 3 to 4 toxicities were fatigue, hypertension, and diarrhea. Furthermore, they showed the changes of growth factors in plasma after cediranib (e.g., bFGF, VEGF receptor 1, and matrix metalloproteinase-2), which were associated with treatment response or survival in this therapy. Based on these promising results, the authors conducted a phase III study of cediranib for 325 patients with recurrent glioblastoma, and the preliminary results were reported at the 2010 SNO annual meeting [33]. Patients were assigned on a 2 : 2 : 1 ratio to cediranib monotherapy 30 mg/day, combination of cediranib 20 mg/day plus lomustine, and lomustine monotherapy plus placebo groups. The 6-month PFS was 16% in cediranib monotherapy, 34.5% in the combination, and 25.8% in lomustine plus placebo groups, respectively, although the results were not significantly different between these groups. The efficacy of cediranib monotherapy seems to be less than the initial phase II study, and the possible reason for this discrepancy is that different doses of cediranib were used between two studies.

Sorafenib and sunitinib are inhibitors of multiple receptor tyrosine kinases including VEGF receptor. Sorafenib was approved by the FDA for the treatment of advanced renal cell carcinoma and hepatocellular carcinoma [100]. Hainsworth et al. conducted a phase II trial of concurrent radiotherapy and temozolomide followed by adjuvant sorafenib and temozolomide for 47 newly diagnosed glioblastomas [80]. This regimen was well tolerated without significant grade 3 or 4 toxicities, although median overall survival and PFS were 12 months and 6 months, respectively. The authors concluded that the addition of sorafenib did not appear to improve the prognosis of these patients. Sunitinib was reported in a phase II study of 21 recurrent malignant gliomas [81]. No objective responses were detected, and median overall survival and PFS were 3.8 and 1.6 months, respectively. This study showed that single-agent sunitinib had insufficient activity for recurrent malignant gliomas.

Vatalanib is a small molecule inhibitor of VEGF receptor, PDGF receptor, and c-kit. In a phase I trial, vatalanib was added to the standard regimen of temozolomide and radiotherapy for 19 newly diagnosed glioblastomas [82]. Response rate was 13%, and median overall survival was 16.2 months, respectively. Pazopanib is a multitargeted tyrosine kinase inhibitor, including VEGF receptor-1, -2, and -3. A phase II trial of pazopanib was performed for recurrent glioblastoma [83]. However, this drug did not have enough antitumor activity with response rate of 5.7% and median PFS of 3.0 months.

Despite several trials of VEGF receptor tyrosine kinase inhibitors, the efficacy has not been established. In a retrospective study of glioblastoma patients who failed VEGF receptor tyrosine kinase inhibitors, bevacizumab salvage therapy still provided benefits with response rate of 21% and 6-month PFS of 12.5%, respectively [101]. Although there are no comparative studies, VEGF receptor inhibition therapy may be less effective for malignant gliomas when compared with bevacizumab [102]. 

### 9.4. PDGF Receptor Tyrosine Kinase Inhibitor (Imatinib, Dasatinib, and Tandutinib)

The PDGF pathway also plays a role in angiogenesis [103]. PDGF receptor inhibitors (e.g., imatinib, dasatinib, and tandutinib) have been performed in clinical trials of malignant gliomas. Imatinib is a multitargeted tyrosine kinase inhibitor and blocks PDGF receptor *α*, PDGF receptor *β*, and c-KIT receptor. Preclinical study has demonstrated the antitumor effect of imatinib on glioblastoma cell lines [104]. A phase II study of imatinib was performed for 112 recurrent gliomas [84]. The 6-month PFS was 16% in glioblastoma, 4.0% in pure/mixed anaplastic oligodendrogliomas, and 9% in low-grade or anaplastic astrocytoma. In 31 glioblastoma patients, response rate was 6%, and median survival was 5.2 months, respectively. This study indicated that single agent imatinib was well tolerated but had limited antitumor activity. A randomized phase III study was conducted for 240 recurrent glioblastoma patients treated by hydroxyurea with or without imatinib [85]. The results from the two arms were very similar, and 6-month PFS was 5% in the combination arm and 7% in the imatinib alone arm, respectively. The authors concluded that there were no clinical benefits from the addition of imatinib. Taken together, these results suggest that imatinib is discouraged in recurrent glioblastoma patients.

Dasatinib and tandutinib are oral molecule inhibitors of several targets, including PDGF and c-kit. Dasatinib was approved by FDA for the treatment of chronic myelogenous leukemia [105]. A retrospective study reported the efficacy of dasatinib for 14 recurrent glioblastomas who failed bevacizumab therapy [86]. However, objective response rate was 0%, and 6-month PFS was 0%, respectively. Currently, a phase II trial of dasatinib (RTOG 0627) is ongoing to evaluate the efficacy and safety for recurrent glioblastoma or gliosarcoma [106]. Combined treatments with tandutinib and bevacizumab are being performed in a phase II study for recurrent malignant gliomas [107]. Preliminary results cautioned that neuromuscular junction dysfunction was observed in this regimen.

Although PDGF receptor inhibitors are effective in preclinical studies, it is still unclear whether these drugs have an antitumor effect in malignant glioma patients. One possible reason for the limited antitumor effect is that PDGF receptor inhibitor such as imatinib cannot cross the blood-brain barrier via the P-glycoprotein efflux pump [108]. 

## 10. Summary and Perspectives

Despite advances in treatment therapeutics, patients with malignant gliomas still have poor prognosis. A better understanding of tumor angiogenesis has allowed us to target VEGF in antiangiogenic therapy. Bevacizumab is considered as a well-established antiangiogenic therapy in several solid tumors. A phase II trials of recurrent glioblastoma showed favorable response rates (28% to 57%) and 6-month PFS (29% to 50.3%) [11–13]. Based on these promising results, bevacizumab was approved by the FDA for the recurrent glioblastoma. Regarding recurrent anaplastic gliomas, bevacizumab has been reported to be effective as well [28, 29]. Additional therapies (e.g., chemotherapy, targeting therapy, and radiotherapy) to bevacizumab have been reported for recurrent malignant gliomas, and these results were encouraging. However, the timing, dosing, and the ideal treatment partners of bevacizumab have remained controversial. Further investigations are warranted to establish an antiangiogenic treatment for recurrent malignant gliomas.

 Bevacizumab is expected to be on the frontline treatment of patients with glioblastoma. Phase II trials have reported the addition of bevacizumab to standard temozolomide and radiotherapy regimen for newly diagnosed glioblastoma [14, 15]. However, the authors concluded that this regimen improved PFS but not overall survival when compared with control group [14]. Currently two randomized phase III trials, RTOG 0825 and AVAGLIO, are ongoing to demonstrate the efficacy and safety of combined therapy of bevacizumab, temozolomide, and radiotherapy for newly diagnosed glioblastoma [56, 57]. These studies will show the role of bevacizumab in the first-line treatment of newly diagnosed glioblastoma.

Many other antiangiogenic therapies (e.g., cilengitide and cediranib) have also been performed in clinical trials. These studies showed encouraging results and are expected to improve the prognosis of malignant gliomas. However, some phase II trials have several limitations such as small sample size, possible enrollment bias, patient selection, and reliance on historical control data. These limitations are associated with a high false-positive rate, and the results from phase II studies are often not validated in phase III studies [109]. Phase II studies must be appropriately planned to have the greatest potential for informing the design of phase III trials [109].

 Antiangiogenic therapies provide favorable results and seem to be attractive strategy in malignant gliomas. However, several problems such as including severe toxicities, resistance, evaluation of response, and lack of predictive biomarkers still remain. The unique severe adverse effects related to bevacizumab have been reported, such as CNS hemorrhage, deep venous thrombosis, and pulmonary emboli [11–13]. BRAIN study reported that Grade ≥3 adverse events were observed 65.8% and 46.4% in bevacizumab plus irinotecan and bevacizumab alone groups, respectively [13]. To reduce the serious adverse events associated with bevacizumab, selecting appropriate patients, early assessment of toxicity, and adequate management should be required.

Malignant glioma patients maintained on bevacizumab inevitably experience the treatment failure. Recurrent tumors following bevacizumab failure appear to be more aggressive with rebound edema [38]. Preclinical study showed that other angiogenic factors, such as fibroblast growth factors and PDGF, can compensate for the loss of VEGF activity under bevacizumab treatment [42]. A novel therapeutic strategy is required to overcome the resistance to bevacizumab of malignant gliomas.

An accurate assessment of tumor response by conventional modality is limited in antiangiogenic therapy due to alterations in tumor blood vessels [44]. The Response Assessment in Neuro-Oncology Working Group proposed that T2 and FLAIR changes on MRI should include the response criteria [47]. FDG-FLT is also expected to accurately evaluate the treatment response in bevacizumab due to its ability to detect cell proliferation [48].

Tumor VEGF expressions or circulating markers potentially predict the prognosis in malignant glioma treated by bevacizumab, although the rational biomarker has not been established. Novel biological markers are required to investigate, providing a personalized treatment that selects the patients who can benefit from bevacizumab.

Although several limitations on antiangiogenic therapy have been reported, this treatment is expected to improve the prognosis of malignant gliomas. Further investigation is warranted to establish the safe and effective antiangiogenic therapy for malignant gliomas. 

## Figures and Tables

**Table 1:** Bevacizumab for recurrent or newly malignant gliomas.

Study	Agents	Patients	RR	MPFS	6-PFS	MST	Ref.
Phase II	Bevacizumab + irinotecan	35 recurrent GBM	57%	6 months	46%	10.5 months	[11]
Phase II	Bevacizumab	85 recurrent GBM	28%	4.2 months	43%	9.2 months	
	Bevacizumab + irinotecan	82 recurrent GBM	38%	5.6 months	50%	8.7 months	[12]
Phase II	Bevacizumab	48 recurrent GBM	35%	4 months	29%	7.7 months	[13]
Phase II	Bevacizumab + irinotecan	23 recurrent GBM	61%	5.0 months	30%	10 months	
		9 recurrent AG	67%	7.5 months	56%	Not reached	[28]
Phase II	Bevacizumab + irinotecan	33 recurrent AG	61%	7.5 months	55%	16.3 months	[29]
Retrospective	Bevacizumab + SRT	20 recurrent GBM	50%	7.3 months	65%	12.5 months	[30]
		5 recurrent AG	60%	7.5 months	60%	16.5 months	
Retrospective	SRS + bevacizumab		—	5.2 months	—	11.2 months	[31]
	SRS + other drugs	49 recurrent GBM	—	2.1 months	—	3.9 months	
Phase II	Bevacizumab + erlotinib	25 recurrent GBM	48%	4.5 months	28%	10.5 months	
		32 recurrent AG	31%	5.9 months	44%	17.8 months	[32]
Phase II	Bevacizumab + RT/TMZ	70 newly diagnosed GBM	—	13.6 months	88%	19.6 months	[14]
Phase II	Bevacizumab + RT/TMZ	125 newly diagnosed GBM	—	13.8 months	87%	—	[15]
Phase II	Adjuvant bevacizumab	125 newly diagnosed					
	+ irinotecan + TMZ	GBM	—	13.8 months	—	21.3 months	[33]

RR: response rate; MPFS: median progression-free survival; 6-PFS: 6-month progression-free survival; MST: median overall survival time; GBM: glioblastoma multiforme; AG: anaplastic gliomas; SRT: stereotactic radiotherapy; SRS: stereotactic radiosurgery; RT: radiotherapy; TMZ: temozolomide.

**Table 2:** Other antiangiogenesis drugs for recurrent or newly diagnosed malignant gliomas.

Target	Study	Agent	Patients	RR	MPFS	6-PFS	MST	Ref.
Integrin		Cilengitide (500 mg/day)	41 recurrent GBM	5%	7.9 months	10%	6.5 months	
	II	(2000 mg/day)	40 recurrent GBM	13%	8.1 months	15%	9.9 months	[73]
Integrin	I/IIa	Cilengitide + RT/TMZ	52 Newly diagnosed GBM	—	8.0 months	69%	16.1 months	[74]
bFGF	II	Thalidomide	39 recurrent MG	6%	2.5 months	—	7.0 months	[75]
bFGF	II	Thalidomide + carmustine	40 recurrent MG	24%	3.3 months	28%	—	[76]
bFGF	II	Thalidomide + irinotecan	32 recurrent GBM	6%	3.3 months	25%	9.0 months	[77]
bFGF	I	Lenalidomide	24 recurrent GBM	0%	1.8 months	13%	6.0 months	[78]
VEGFR	II	Cediranib (45 mg/day)	31 recurrent GBM	27%	3.9 months	26%	7.6 months	[79]
		Cediranib (30 mg/day)	325 recurrent GBM	—	—	16%	—	
VEGFR	III	Cediranib (20 mg/day) + lomustine		—	—	35%	—	[33]
		Lomustine + placebo		—	—	26%	—	
VEGFR	II	Adjuvant sorafenib + TMZ	47 newly diagnosed GBM	13%	6.0 months	50%	12 months	[80]
VEGFR	II	Sunitinib	21 recurrent MG	0%	1.6 months	—	3.8 months	[81]
VEGFR	I	Vatalanib + RT/TMZ	19 newly diagnosed GBM	13%	7.2 months	—	16.2 months	[82]
VEGFR	II	Pazopanib	35 recurrent GBM	6%	3.0 months	3%	8.8 months	[83]
PDGFR	II	Imatinib	31 recurrent GBM	6%	1.7 months	16%	5.2 months	[84]
PDGFR		Imatinib	120 recurrent GBM	—	1.5 months	7%	5.3 months	
	III	Imatinib + hydroxyurea	120 recurrent GBM	—	1.5 months	5%	4.8 months	[85]
PDGFR	R	Dasatinib	14 recurrent GBM	0%	0.9 months	0%	2.6 months	[86]

RR: response rate; MPFS: median progression-free survival; 6-PFS: 6-month progression-free survival; MST: median overall survival time; GBM: glioblastoma multiforme; RT: radiotherapy; TMZ: temozolomide; bFGF: basic fibroblast growth factor; MG: malignant gliomas; VEGFR: vascular endothelial growth factor receptor; PDGFR: platelet-derived growth factor receptor; R: retrospective.
